# DNA Damage Repair and Drug Efflux as Potential Targets for Reversing Low or Intermediate Ciprofloxacin Resistance in *E. coli* K-12

**DOI:** 10.3389/fmicb.2018.01438

**Published:** 2018-07-02

**Authors:** Rasmus N. Klitgaard, Bimal Jana, Luca Guardabassi, Karen L. Nielsen, Anders Løbner-Olesen

**Affiliations:** ^1^Department of Biology, Section for Functional Genomics, University of Copenhagen, Copenhagen, Denmark; ^2^Department of Veterinary and Animal Sciences, Section for Veterinary Clinical Microbiology, University of Copenhagen, Copenhagen, Denmark; ^3^Department of Clinical Microbiology, Center for Diagnostics, Rigshospitalet, Copenhagen, Denmark

**Keywords:** antibiotic resistance, ciprofloxacin, helper drugs, RNA-Seq, transcriptomics

## Abstract

Ciprofloxacin is a potent antibacterial drug that is widely used in human clinical applications. As a consequence of its extensive use, resistance has emerged in almost all clinically relevant bacterial species. A mean to combat the observed ciprofloxacin resistance is by reversing it via co-administration of a potentiating compound, also known as a helper drug. Here, we report on the current advances in identifying ciprofloxacin helper drugs, and put them into perspective of our own findings. We searched for potential helper drug targets in *Escherichia coli* strains with different levels of ciprofloxacin resistance using transcriptomics i.e., RNAseq and by deletion of genes associated with hyper-susceptibility to ciprofloxacin. Differential gene expression analysis of the highly ciprofloxacin resistant uropathogenic *E. coli* strain, ST131 UR40, treated with a clinically relevant concentration of ciprofloxacin (2 μg/mL), showed that the transcriptome was unaffected. Conversely, genetic screening of 23 single gene deletions in the high-level ciprofloxacin resistant laboratory derived *E. coli* strain, LM693, led to a significant decrease in the minimal inhibitory concentration for several genes, including genes encoding the AcrAB-TolC efflux pump, SOS-response proteins and the global regulator Fis. In addition, deletion of *acrA*, *tolC*, *recA*, or *recC* rendered two *E. coli* strains with intermediate susceptibility to ciprofloxacin fully susceptible according to the CLSI recommended breakpoint. Our results corroborate the AcrAB-TolC efflux pump and the SOS response proteins, RecA and RecC, as potential targets for ciprofloxacin helper drugs in treatment of human bacterial infections caused by *E. coli* strains with intermediate sensitivity to ciprofloxacin.

## Introduction

Fluoroquinolones are some of the most prescribed antibacterial drugs in the world, commonly used for the treatment of urinary tract infections and sinusitis (Emmerson and Jones, 2003; Linder et al., 2005; Mitscher, 2005), but this has not always been the case. For the first two decades after the discovery of nalidixic acid in 1962, and its introduction into the clinic in 1964, the quinolones were only used to treat uncomplicated urinary tract infections. This changed with the release of the second generation quinolones, including ciprofloxacin, which showed significant activity outside the urinary tract and against a broad spectrum of both Gram-negative and Gram-positive bacteria. Ciprofloxacin acts by binding to its targets, DNA gyrase and topoisomerase IV, inhibiting the native ability of these two enzymes to re-ligate double stranded DNA breaks, in turn leading to fragmentation of the chromosome. Due to its mechanism of action it is sometimes referred to as *topoisomerase poison* (Aldred et al., 2014). Inevitably, considering its extensive use and misuse, resistance toward ciprofloxacin has increased in almost all clinically relevant bacteria (Werner et al., 2011; Dalhoff, 2012). One method to overcome antibacterial resistance is by combinatorial treatment with a potentiating compound, also known as a helper drug. A helper drug is by definition non-antibacterial when administered alone, but it enhances the activity of the antibiotic when used in concert. The potentiating effect of a helper drug can be achieved by either direct inhibition of the resistance mechanism or by targeting endogenous cellular components and pathways like, cell membranes, efflux pumps and cellular repair systems. A classic example of targeting the resistance mechanism is the combination of amoxicillin and the β-lactamase inhibitor clavulanic acid (White et al., 2004). High-level ciprofloxacin resistance is primarily associated with multiple target site mutations in *gyrA* and *parC*, encoding subunits of the DNA gyrase and topoisomerase IV, respectively (Aldred et al., 2014). Since 1998 three different plasmid-mediated ciprofloxacin resistance mechanisms have been identified; (i) target protection (Qnr proteins*)*, (ii) efflux pumps (QepA and OqxAB) and (iii) drug modification (AAC(6′)-Ib-cr acetyltransferase) (Rodríguez-Martínez et al., 2016).

### Potential Ciprofloxacin Helper Drug Targets

Studies of the endogenous cellular mechanisms involved in ciprofloxacin susceptibility and resistance evolution have revealed more than two dozen gene deletions that lead to increased ciprofloxacin susceptibility in wild-type *Escherichia coli* (Cirz et al., 2005; Tamae et al., 2008; Liu et al., 2010; Yamada et al., 2010). Thus, suggesting the gene products as potential ciprofloxacin helper drug targets. Recently, Tran et al. identified 23 single gene deletions that increased the ciprofloxacin susceptibility of a laboratory derived resistant *E. coli* strain. The most significant increase in ciprofloxacin susceptibility was observed for the deletion of SOS-response genes directly involved in DNA damage repair, and the genes encoding the AcrAB-TolC efflux pump (Tran et al., 2016). Recacha et al. recently showed that deletion of *recA* rendered a laboratory derived *E. coli* strain with intermediate sensitivity to ciprofloxacin clinically susceptible *in vitro.* In addition, the *in vivo* efficacy of ciprofloxacin against the same strain was significantly increased in a peritoneal sepsis murine model (Recacha et al., 2017). Thus, the current evidence suggests that targeting the repair of ciprofloxacin induced DNA damage or the efflux pump AcrAB-TolC are the most promising strategies for ciprofloxacin helper drugs. Here, we used a combined transcriptomic and genetic approach in an attempt to both identify novel helper drug targets, as well as further assess the potential of known helper drug targets in laboratory derived *E. coli* strains with different levels and mechanisms of ciprofloxacin resistance.

## Materials and Methods

### Bacterial Strains and Plasmids

Strains LM693 and LM862 were obtained from Diarmaid Hughes from Uppsala University. LM693 is isogenic to the commonly used laboratory strain, MG1655, besides two *gyrA* mutations, S83L and D87N, and one parC mutation, S80I. LM862 is also isogenic to MG1655, but with one *gyrA* S83L mutation and one *parC* S80I mutation. ST131 UR40 has two *gyrA* mutations, S83L and D87, and two parC mutations, S80I and E84V, and carries *aac-6′-Ib-cr* on a plasmid (Cerquetti et al., 2010). The *aac-6′-Ib-cr* carrying plasmid pRNK1 (was constructed as follows: *aac-6′-Ib-cr* gene was amplified by PCR from ST131 UR40, using the following primers: GATCGGATCCATGAGCAACGCAAAAACAAAGTTAGGC and CATCGAATTCTTAGGCATCACTGCGTGTTCGC, and cloned into pMW119 (Nippon Gene, Toyama, Japan) using *Bam*HI and *Eco*RI. The *qnrS*-carrying plasmid pRNK9 was constructed as follows: *qnrS* was amplified by PCR from the clinical *E. coli* isolate EC38 using the following primers: GATCGGATCCATGGAAACCTACAATCATACATATCGGC and GATCAAGCTTTTAGTCAGGATAAACAACAATACCCAGTGC, and cloned into pMG25 using *Bam*HI and *Hin*dIII (M. Mikkelsen and K. Gerdes, unpublished). pRNK1 (4796 bp) and pRNK9 (4723 bp) was then introduced in LM862 by electroporation. Strain EC38 was isolated from a patient with a urinary tract infection at Hvidovre Hospital, Denmark.

### Genetic Screening and MIC Tests

For the genetic screen, P1 phage lysates were prepared from the relevant Keio collection strains (Baba et al., 2006) and used for transduction into LM693 and LM862. All the transduced strains were verified by PCR. The ciprofloxacin MICs for LM693 and derived strains were determined using E-tests (0.002–32 μg/ml, BioMerieux) and according to the manufacturer’s guidelines. The MICs for LM862 and derived strains were determined by broth micro-dilution using cation adjusted Mueller Hinton broth II with 1 mM and 10 μM IPTG for pRNK1 and pRNK9, respectively. The two different IPTG concentrations were used to obtain a ciprofloxacin MIC for LM862/pRNK1 and LM862/pRNK9 of 2 μg/mL i.e., within the CLSI intermediate susceptible range. The reference *E. coli* strain ATCC 25922 was used as standard in all MIC tests and the susceptibility was evaluated according to CLSI recommended breakpoints.

### Checkerboard Assay

All wells in a micro-titter plate were filled with 100 μl cation adjusted Mueller Hinton broth II (200 μL in the negative control wells). Copper phtalocyanine-3,4′,4″,4‴-tetrasulfonic acid, was added to the first row, followed by serial dilution along the abscissa, leading to a start concentration of 100 μM. Hereafter ciprofloxacin was serial diluted along the ordinate, giving a start concentration of 2 and 64 μg/ml for LM862 and LM693, respectively. Hundred microliter diluted culture with an OD600 of 0.001 was then inoculated in each well and the plates were incubated at 37°C for 24 h.

### RNA-Sequencing

Ciprofloxacin was added to a culture of ST131 UR40, which had been growing exponentially for more than six generations, to a final concentration of 2 μg/ml. Samples for RNA isolation were taken at 0 min (prior to ciprofloxacin addition) and 30 and 90 min after ciprofloxacin addition, which has previously been shown to be long enough to induce fragmentation of the *E. coli* chromosome (Charbon et al., 2014). Total RNA was isolated using a Thermo Scientific GeneJET RNA isolation kit. Dnase treated with TURBO DNA-free kit from Ambion. rRNA was depleted using an Illumina Ribo-zero rRNA removal kit, followed by RNA-Seq library prep using an Illumina TruSeq Stranded mRNA Library Prep Kit. Sequencing was performed on an Illumina Miseq with a Miseq reagent kit v3. (75 bp paired-end) from Illumina. Data analysis was performed in Rockhopper ver.2.03 (McClure et al., 2013). *E. coli* NA114 (ST131) (accession number: NC_017644) was used as reference genome (Avasthi et al., 2011). The percentage of successfully aligned reads varied from 91 to 88% of the total read count. The sequencing data files and the Rockhopper results from the differential gene expression analysis are available from the Gene Expression Omnibus (GEO: GSE89507).

## Results

### Identification of Helper Drug Targets by Genetic Screening

As mentioned in the introduction several single gene deletions are known to increase the ciprofloxacin susceptibility of *E. coli.* To further assess the helper drug target potential of these genes, 23 single gene deletions were introduced into the high-level ciprofloxacin resistant *E. coli* strain LM693 (MIC of 24–32 μg/ml) (Marcusson et al., 2009) and tested for hyper-susceptibility toward ciprofloxacin (**Table 1**). LM693 is isogenic to the commonly used laboratory strain MG1655 besides two GyrA mutations; S83L and D87, and one ParC mutation; S80I. Even though nine of the mutant strains showed a three to four fold reduction in the MIC, none of them were found to be susceptible according to the CLSI MIC breakpoint for ciprofloxacin (≤1 μg/mL). Our results therefore indicate that none of the tested gene-knockouts identify valid helper drug targets in high-level ciprofloxacin resistant *E. coli* strains. However, they could potentially be used as helper drug targets in bacteria with intermediate susceptibility to ciprofloxacin. To create two strains with intermediate ciprofloxacin susceptibility, we constructed the plasmids pRNK1 and pRNK9 carrying the ciprofloxacin resistance determinants aac-6′-Ib-cr and qnrS, respectively. AAC-6′-Ib-cr inactivates ciprofloxacin by *N*-acetylation of the amino nitrogen of its piperazinyl substituent (Robicsek et al., 2006), while QnrS acts as a DNA mimic, binding to and protecting the gyrase from the action of ciprofloxacin (Rodríguez-Martínez et al., 2016). Introduction of pRNK1 and pRNK9 into strain LM862, which carries GyrA S83L and ParC S80I mutations, increased the MIC from 1 to 2 μg/ml, i.e., into the CLSI intermediate susceptible MIC range. We then evaluated the ability of seven of the most promising gene deletions described above to reduce the ciprofloxacin MIC of LM862/pRNK1 and LM862/pRNK9. Four of the gene deletions (*acrA, tolC, recA*, and *recC*) rendered both strains susceptible to ciprofloxacin (**Table 2**). To assess whether inhibition of RecA was an amenable strategy for potentiation of ciprofloxacin, synergy between ciprofloxacin and a RecA inhibitor, copper phtalocyanine-3,4′,4″,4‴-tetrasulfonic acid (Alam et al., 2016), was tested by a checkerboard assay. However, we did not observe a reduction in the ciprofloxacin MICs for either LM693 or LM862.

**Table 1:** MIC values for the single gene deletions in LM693.

Strain/single deletions	MIC (μg/ml)
LM693	24–32
*tolC*	1.5
*acrA, acrB* and *fis*	2
*recC, xseA, xseB, uvrD*, and *recA*	4
*ruvC* and *dksA*	6
*recG* and *hlpA*	8
*pgm, ybgF and ybgC*	12
*deoR, ydcS, yciT and ybjQ*	16
*ygcO* and *nlpC*	24
*rimK*	24–32


**Table 2:** MIC values for the single gene deletions in LM862/pRNK1 and LM862/pRNK9.

Strain	MIC (μg/ml)	
LM862	1	1
LM862/Empty vectors	1	1
	**pRNK1**	**pRNK9**
LM862	2	2
*tolC*	0.25	0.5
*acrA*	0.25	0.5
*recA*	0.5	0.5
*recC*	0.5	0.5
*uvrD*	2	1
*xseA*	1	1
*fis*	2	4


### Identification of Helper Drug Targets by RNA Sequencing

The *E. coli* clonal group ST131 has become the predominant *E. coli* lineage isolated from human extra-intestinal infections and is currently regarded a global problem in hospitals and clinical practices (Nicolas-Chanoine et al., 2014). Two independent studies have shown that more than 90% of ESBL-producing ST131 isolates are also resistant to ciprofloxacin (Brisse et al., 2012; López-Cerero et al., 2014). Strain ST131 UR40 is resistant to high levels of ciprofloxacin due to GyrA mutations S83L and D87, and ParC mutations S80I and E84V (Cerquetti et al., 2010). Here we used RNA-Seq to map the transcriptomic changes during treatment of ST131 UR40 with a clinically relevant concentration of ciprofloxacin, 2 μg/ml, which is approximately equal to the maximum serum concentration following oral administration of 500 mg ciprofloxacin according to the FDA. The rationale behind this was to identify potential helper drug target genes that were upregulated upon ciprofloxacin exposure and thereby potentially involved in ciprofloxacin resistance. In contrast to the genetic screen, the RNA-Seq analysis would also reveal targets encoded by essential genes and non-coding RNA. The transcriptomic analysis did not show any non-ribosomal transcripts to be significantly upregulated in the presence of ciprofloxacin, i.e., with a false discovery rate of <1% and more than two-fold expression change.

## Discussion

By utilizing a combination of “direct genetic screening” and differential gene expression analysis, we have attempted to identify potential genes suitable as targets for ciprofloxacin potentiating compounds. We did not find any genes to be significantly upregulated by ciprofloxacin, indicating that the transcriptome of ST131 UR40 was relatively unaffected by treatment with a sub-inhibitory and yet clinically relevant concentration of ciprofloxacin. The lack of an upregulation of the SOS response genes in the transcriptomic analysis suggests that the ciprofloxacin exposure did not cause sufficient DNA damage to induce a SOS response; hence it was not necessary for ST131 UR40 to upregulate any specific genes to cope with the presence of ciprofloxacin at a sub-inhibitory concentration.

The screening of selected mutant strains revealed a number of genes, which when deleted, lowered the MIC for ciprofloxacin significantly in LM693. These findings are in accordance with genes reported to contribute to high-level ciprofloxacin resistance by Tran et al. (2016). Treatment of bacteria with ciprofloxacin generates double stranded breaks in the DNA of the organism (Drlica et al., 2008), which in turn activates the SOS response. Seven of the tested gene deletions; *recA, recC, recG, uvrD, xseAB*, and *ruvC*, which all significantly reduced the MIC of LM693, are part of the SOS response and involved in DNA damage repair (Chase and Richardson, 1974; Kuzminov, 1993; Michel, 2005). Thus, deletion of any of these seven genes likely lowers the ability of the bacteria to cope with ciprofloxacin induced DNA damage. Deletion of genes encoding the AcrAB-TolC efflux pump, or the global regulator Fis (Factor for inversion stimulation) showed the largest decreases in MIC values for LM693. The Fis protein has been shown to repress the *gyrA* and *gyrB* promoters, thereby reducing the expression of the DNA gyrase (Schneider et al., 1999). Thus, deletion of *fis* likely increases DNA gyrase expression and the number of ciprofloxacin targets. As ciprofloxacin works as a topoisomerase poison, an increase in ciprofloxacin bound DNA gyrase could potentially lead to an increase in double stranded breaks, explaining the decrease in MIC for the *fis* deletion strain. The *fis* deletion did not have the same effect on the intermediate susceptible strains LM862/pRNK1 and LM862/pRNK9, which may be explained by the relatively higher affinity of ciprofloxacin for its target in LM862, compared to that of LM693. Thus, the increase in expression of the DNA gyrase might lead to an increase in ciprofloxacin-gyrase complexes, but if the ciprofloxacin induced DNA damage is already at a level, where the DNA repair mechanisms cannot keep up, the *fis* deletion does not have a dramatic effect on the MIC.

Individual deletions of *acrA, acrB*, or *tolC* genes encoding the AcrAB-TolC efflux pump had a large effect on the ciprofloxacin susceptibility of both LM693 and LM862 strains. This was not surprising as overexpression of the AcrAB-TolC efflux system has been connected to ciprofloxacin resistance numerous times (Mazzariol et al., 2000). The deletion of *acrA* or *tolC* in the LM862 strains lowered the MIC beneath the CLSI susceptible breakpoint indicating that AcrAB-TolC efflux system is a potential target for ciprofloxacin potentiating compounds in intermediate susceptible *E. coli*. A number of AcrAB-TolC inhibitors have been identified (Chevalier et al., 2004; Bohnert et al., 2013; Aparna et al., 2014; Opperman et al., 2014; Yilmaz et al., 2015), two of which have been shown to decrease the MIC of ciprofloxacin in susceptible *E. coli* strains (Opperman et al., 2014; Yilmaz et al., 2015), but none of them are used in clinical practice so far.

Inhibition of RecA and thereby of the SOS response has been proposed as a strategy to fight antibiotic resistance numerous times (Blázquez et al., 2012; Culyba et al., 2015; Alam et al., 2016). Our finding, that deletion of *recA* render intermediate susceptible strains of *E. coli* fully susceptible to ciprofloxacin is in accordance with recent observations by Recacha et al. (2017). Overall, this indicates that RecA could be a potential ciprofloxacin helper drug target.

Even though AcrAB-TolC or RecA deficiency rendered LM862/pRNK1 and LM862/pRNK9 susceptible to ciprofloxacin, the respective MICs were only two to four-folds lower than the susceptible MIC breakpoint. It therefore seems reasonable to assume that a given inhibitor should completely block the activity of either RecA or AcrAB-TolC in order for it to be an efficient helper drug. This hypothesis is backed by the failure of lowering the ciprofloxacin MIC of LM862 and LM693 with the RecA inhibitor phtalocyanine-3,4′,4″,4‴-tetrasulfonic acid.

## Conclusion

The findings of this study and the evidence given in the literature, indicates that reversal of ciprofloxacin resistance in high-level resistant *E. coli* strains by the use of helper drugs does not appear to be plausible. Conversely, targeting RecA, RecC or the AcrAB-TolC efflux pump is a likely feasible strategy for reversing ciprofloxacin resistance in *E. coli* strains with intermediate susceptibility to ciprofloxacin. However, it should be noted that there is a discrepancy between the MIC breakpoint for ciprofloxacin susceptibility proposed by the CLSI (≤1 μg/mL) and the EUCAST (≤0.25 μg/mL). Therefore, further *in vivo* studies are needed to asses if targeting either RecA, RecC, or AcrAB-TolC leads to a significant increase in the efficacy of ciprofloxacin against an intermediate susceptible *E. coli* strain.

## Author Contributions

RK carried out all experimental work, designed the study, analyzed the data, and prepared the final manuscript. AL-O supervised all aspects of the study and helped prepare the final manuscript. BJ assisted and supervised the experimental part of the RNA-seq. LG supervised and delivered the ST131 UR40 strain. KN performed genomic analyses and delivered the EC38 strain carrying the *qnrS* gene. All authors read and approved the final manuscript.

## Conflict of Interest Statement

The authors declare that the research was conducted in the absence of any commercial or financial relationships that could be construed as a potential conflict of interest.

## Abbreviations

MIC: minimal inhibitory concentration
ST: sequence type
